# Biogenic Silver Nanoparticles as a Post-surgical Treatment for *Corynebacterium pseudotuberculosis* Infection in Small Ruminants

**DOI:** 10.3389/fmicb.2019.00824

**Published:** 2019-04-24

**Authors:** Laerte Marlon Santos, Danijela Stanisic, Ulisses José Menezes, Marcos Antônio Mendonça, Thiago Doria Barral, Núbia Seyffert, Vasco Azevedo, Nelson Durán, Roberto Meyer, Ljubica Tasic, Ricardo Wagner Portela

**Affiliations:** ^1^Laboratório de Imunologia e Biologia Molecular, Instituto de Ciências da Saúde, Universidade Federal da Bahia, Salvador, Brazil; ^2^Laboratório de Química Biológica, Departamento de Química Orgânica, Instituto de Química, Universidade Estadual de Campinas, Campinas, Brazil; ^3^Programa de Pós-Graduação em Microbiologia, Instituto de Biologia, Universidade Federal da Bahia, Salvador, Brazil; ^4^Laboratório de Genética Celular e Molecular, Instituto de Ciências Biológicas, Universidade Federal de Minas Gerais, Belo Horizonte, Brazil; ^5^Laboratório de Biologia Estrutural e Funcional, Instituto de Biologia, Universidade Estadual de Campinas, Campinas, Brazil

**Keywords:** antimicrobials, caseous lymphadenitis, nanotechnology, small ruminants, wound healing

## Abstract

Caseous lymphadenitis (CL) is an infectious and zoonotic disease characterized by the development of granulomas in the lymph nodes and internal organs of small ruminants. The etiological agent of this disease is *Corynebacterium pseudotuberculosis*, a Gram-positive and facultative intracellular bacterium. The conventional treatment for CL consists of drainage and chemical cauterization of the lesions using a 10% iodine solution. However, this type of treatment is not effective, due to iodine’s cytotoxic profile and low antibacterial activity. Currently, silver nanoparticles (AgNPs) can be seen as an alternative treatment for CL due to their antimicrobial activity and wound healing effects. Therefore, the present study aimed to evaluate AgNPs as a post-surgical treatment for CL. Twenty-nine goats and sheep with clinical signs of CL were selected. Surgical intervention was performed to excise the caseous lesions. To treat the lesions, an ointment formulation based on AgNP mixed with natural waxes and oils was used in the experimental group, and the conventional treatment with 10% iodine was used in the control group. Bacteria were isolated from the excised caseous material. The animals were observed for 8 weeks after the surgical treatment, and blood samples were taken weekly. The surgical wounds of sheep treated with AgNP healed faster, and the surgical wound area was smaller during the observation period; the latter effect was also observed in goats. AgNP-treated animals also had less purulent discharge and less moisture in the surgical wounds. The AgNP-treated animals had lower leukocyte counts and lower titers of anti-*C. pseudotuberculosis* antibodies. There was no statistically significant difference between the groups with regard to the hemogram results. The results of the susceptibility testing of *C. pseudotuberculosis* strains (T1, 1002, FRC41, and VD57 strains) and clinical isolates to AgNPs showed growth inhibition, even at low concentrations. It can be concluded that post-surgical treatment of CL using the AgNP-based ointment may be a promising tool in the control of CL, through faster healing, decreased wound contamination, and no apparent toxic effects.

## Introduction

Caseous lymphadenitis (CL), an infectious disease caused by the bacterium *Corynebacterium pseudotuberculosis* that affects small ruminants, has a chronic and debilitating profile and a zoonotic potential. CL is characterized by the development of granulomas in the lymph nodes and in organs such as the spleen, lungs, liver, and kidneys (Dorella et al., 2006). The disease is associated with a decrease in the production of wool and meat, as well as a high cost of treatment (Santiago et al., 2013).

A definitive diagnosis of CL is made through the isolation of the etiologic agent from caseous material retrieved from lesions. The only possible treatment currently conducted in sheep and goat farms is drainage of the lesions with an application of a 10% iodine solution for chemical cauterization (de Farias et al., 2018), a procedure that can hinder the healing process due to the histotoxic profile of iodine (Punjataewakupt et al., 2019). The secretion from the operatory wounds pose a potential risk for environmental contamination since *C. pseudotuberculosis* can survive for long periods in the soil at sheep and goat farms (Spier et al., 2012).

The inappropriate use of antimicrobials has been a cause of multidrug-resistant bacteria development. In response, conventional antibiotics are being substituted by new alternative technologies such as nanotechnology, which has broad potential use in human and veterinary medicine (Rudramurthy et al., 2016). Silver nanoparticles (AgNPs) are seen as a good option among nanoparticles with antimicrobial activity because, besides having a marked antibacterial profile, they also contribute to wound healing, are durable and efficacious, and are relatively inexpensive to produce (Lee et al., 2007; Zang et al., 2008).

It is necessary that new compounds be developed for the post-surgical treatment of CL. Given the antibacterial activity and wound healing effects of AgNP, this study aimed to verify the efficacy of a biogenic AgNP topical ointment in the treatment of sheep and goats who underwent extraction of CL lesions.

## Materials and Methods

### Animals and Ethical Aspects

This study was carried out in a sheep and goat breeding farm in the municipality of Capim Grosso, Bahia State, Brazil. Twenty-nine mixed breed animals with clinical signs of CL were selected. These animals had a complete surgical removal of the CL lesions with a standardized incision size and were divided into four groups: group CP (10 goats treated with AgNP ointment); group CI (10 goats treated with 10% iodine solution); group OP (5 sheep treated with the AgNP-based ointment); and group OI (4 sheep treated with 10% iodine solution). All the animals were treated at the time of the CL lesion excision. The clinical, serological, hematological, and biochemical follow-up was carried out shortly before the surgical procedure and within 8 weeks after the surgical procedure. This study was approved by the Committee on the Use of Animals in Scientific Experimentation of the School of Veterinary Medicine of the Federal University of Bahia (protocol number 35/2017).

### Biogenic AgNP Synthesis

The synthesis of the AgNPs was performed following the procedure described by Ballottin et al. (2017). Briefly, *Fusarium oxysporum* fungus was grown in a solid culture medium consisting of 0.5% yeast extract, 2% malt, 2% agar and distilled water, and kept at 28°C for 1 week. After growth, sterilized distilled water was added to the culture under constant stirring until reaching a protein concentration of 0.1 g/mL and kept under stirring for 72 h. A vacuum filtration was performed and then 0.01 mol/L of AgNO_3_ was added to the supernatant. The solution was maintained at 28°C and sealed with aluminum foil until the formation of the nanoparticles. The AgNPs were characterized and showed sizes of 28.0 ± 13.1 nm, polydispersity of 0.231, zeta potential of −31.7 ± 2.8 mV, and were spherical in form (Stanisic et al., 2018).

### AgNP Ointment Production

For the post-surgical treatment of CL, an ointment based on AgNPs, natural waxes, and oils was made, as described by Stanisic et al. (2018). In the first step of ointment production, the oils and waxes (31% solid vaseline, 20% lanoline, 10% liquid vaseline, 9.0% cera alba, 8.0% cetostearyl alcohol, and 2.0% cholesterol) were blended and heated at 60°C until the mixture was homogeneous, and all of the ingredients were fused. Then, the emulsion was cooled to room temperature (25°C), while constantly mixing to maintain its homogeneity. In the second step of ointment production, 20% part-to-part of a 12.8 mg/mL colloidal solution of biogenic AgNPs was added gradually to the mixture at room temperature under constant stirring (Stanisic et al., 2018). The final ointment was a gray oil/water cream that was easy to apply and left an oily film when applied to the skin surface.

### Bacterial Strains

For the *in vitro* analysis of bacterial susceptibility to AgNPs, we used four *C. pseudotuberculosis* strains: the 1002 strain, which is used as a reference in the genome project of the bacteria (Mariano et al., 2016) and has already had its whole genome sequenced (GenBank CP001809.2); the VD57 strain, which is a highly virulent strain isolated from a goat CL lesion (Almeida et al., 2016) that has a sequenced genome (GenBank CP009927.1); the FRC41 strain, which was isolated from a human case of lymphadenitis in France (Join-Lambert et al., 2006), and also has a sequenced genome (GenBank CP013146.1); and the T1 strain, which is an attenuated strain used as a CL vaccine model (Moura-Costa et al., 2008), and also has a sequenced genome (GenBank CP015100.1). All the *C. pseudotuberculosis* strains herein used belong to the biovar *ovis*.

### Isolation of *C. pseudotuberculosis* From Caseous Lesions

For the isolation of *C. pseudotuberculosis* from the granuloma material, caseous samples collected from the animals that underwent the CL lesion excision were inoculated into Brain and Heart Infusion agar (HIMEDIA, Mumbai, India) and supplemented with 5% sheep blood. After inoculation, the plates were incubated for 48 h at 37°C. The isolated colonies were macroscopically characterized, stained using the Gram method, and subjected to biochemical tests. Colonies that were Gram-positive, exhibited catalase production, demonstrated positive reactions in the glucose, maltose, sucrose, and urea tests, and negative reactions in the lactose, trealose, salicin, and motility tests were identified as *C. pseudotuberculosis* (Moura-Costa et al., 2008).

### Determination of *C. pseudotuberculosis* Laboratorial Strains and Clinical Isolates Susceptibility/Resistance to AgNP Profile

The broth microdilution methodology was performed according to Norman et al. (2014), with modifications. The AgNP solution was diluted in sterile milliQ water in concentrations ranging from 7.5 to 0.02 mg/mL. The strains were inoculated in BHI broth (HIMEDIA, Mumbai, India) Tween 80 0.1% for 12 h prior to the assay. After incubation, the strains were diluted in 2 × BHI to achieve an optical density of 0.08–0.10 at 600 nm, which contains 3 × 10^6^ CFU/mL of *C. pseudotuberculosis*. Subsequently, these suspensions were diluted in BHI broth to obtain a concentration of 1 × 10^6^ CFU/mL. One hundred μL of the inoculum and 100 μL of the AgNP colloidal solution were placed in each well. Two controls were used for each dilution, one positive (bacterial suspension without AgNPs) and one negative (AgNP colloidal solution without bacterial inoculation). Plates were then incubated for 48 h at 37°C. Analysis was performed in a spectrophotometer at the wavelength of 600 nm and the minimal concentration that inhibited 100% of the bacterial growth (MIC_100_) was established. Then, 20 μL of each well was removed and inoculated into BHI agar plates, which were incubated for 48 h at 37°C. Minimal bactericidal concentration (MBC_100_) was defined as the lowest concentration capable of killing all bacteria.

### Surgical Procedure

Before the surgical incision, the hair in the skin above the CL-affected lymph node was shaved, followed by a 70% alcohol asepsis. For the surgical treatment, a 2 cm long standard incision was made with a #4 scalpel blade on the CL lesions with subsequent drainage of all the caseous material. This material was collected in sterile containers and sent to the laboratory to confirm the presence of *C. pseudotuberculosis* through microbiological assays. In CI and OI groups, a commercial 10% iodine solution was used in the surgical wound. In the CP and OP groups, the AgNP ointment was added to the surgical lesion, filling all space left by the drainage of the caseous lesion (Supplementary Figure S1). The clinical, hematological and serological follow-up of the animals were performed immediately before and after the surgical procedure, and within 8 weeks after the surgery, with a 1-week interval between observations and collections. The animals’ cardiac and respiratory rate, rectal temperature, body score, and skin turgor return time were evaluated during the observation period.

Lesion drainage occurred in a location the herd could not access, and, in order to reduce environmental contamination, treated animals only returned to the herd after the lesion had completely healed. Jugular venipuncture was performed just before the surgery and weekly for 8 weeks following surgery. Blood was collected (10 mL) in a Vacutainer^®^-type tube without anticoagulant. Samples were then centrifuged at 4000 ×*g* for 10 min and the serum was obtained. These samples were used in the serological tests. Also, blood samples (10 mL) were collected in Vacutainer^®^ tubes with ethylenediaminetetraacetic acid (EDTA) anticoagulant to perform blood cells counts using an automatized counter. The area of open the surgical wounds were measured using a caliper over the course of the 8 weeks of post-surgical follow-up.

### Immunological Assay

To detect *C. pseudotuberculosis*-specific antibodies in the animals’ serum samples, it was used serum samples collected from the animals during the observation period. The serum samples were analyzed using the indirect ELISAs developed by Rebouças et al. (2013) for sheep and by Seyffert et al. (2010) for goats.

### Statistical Analysis

The statistical analyses were conducted using SPSS 18 (IBM, United States). The unpaired Student *t*-test was used to compare blood cell counts, open wound areas, body and hydration scores, and ELISA results between groups. Data from animals that had moisture and purulent secretion in their surgical wounds and the presence of lymph node enlargement were compared using the chi square test. The curves of bacterial growth inhibition by AgNPs, as well as the surgical wounds healing, were compared using the Wilcoxon signed rank test. Values with *p* < 0.05 were considered statistically significant.

## Results

### Susceptibility of *C. pseudotuberculosis* Strains and Clinical Isolates to AgNP

**FIGURE 1:**
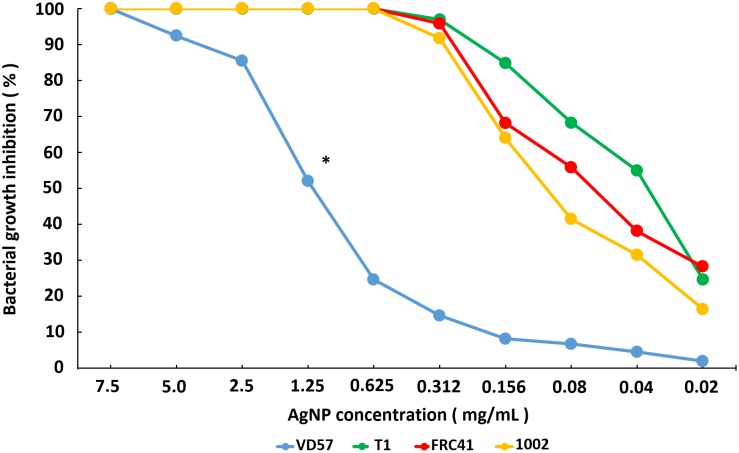
Inhibition of *C. pseudotuberculosis* strains growth by different concentrations of AgNP. The results are expressed as the means of three different experiments. The curves of inhibition were statistically compared using the Wilcoxon signed-rank test, and the symbol “^∗^” indicates statistical difference with *p* < 0.05.

Evaluation of the *C. pseudotuberculosis* standard strain’s susceptibility to AgNP using the broth microdilution method (Norman et al., 2014) indicated a 100% inhibition of the growth of T1, 1002, and FRC41 strains at a 0.625 mg/mL concentration of AgNP. The virulent strain VD57 showed MIC_100_ only at an AgNP dilution of 7.5 mg/mL. Similar results were observed for the minimal bactericide concentration values, with a MBC_100_ of 1.25 to 0.625 mg/mL for the 1002, FRC41, and T1 strains. We observed no bactericidal action for VD57 in the AgNP concentrations used in this experiment (Table 1). When comparing the growth inhibition curves, the 1002, T1, and FRC41 strains demonstrated a similar pattern of inhibition, but were statistically different from the VD57 curve on the Wilcoxon signed rank test (Figure 1).

**Table 1:** Susceptibility of *C. pseudotuberculosis* strains to AgNP.

Host	Strain	MIC_100_ (mg/mL)		MBC_100_ (mg/mL)
Goat	1002	0.625		0.625
Human	FRC41	0.625		1.25
Goat	T1	0.625		1.25
Goat	VD57	7.5		>7.5

*A microdilution in broth assay was carried out with different AgNP dilutions (0.02–7.5 mg/ml). MIC_100_ is defined as the AgNP concentration where there was inhibition of 100% of bacterial growth, and MBC_100_ as the AgNP concentration where there was 100% of bactericidal action, in mg/ml.*

As for the clinical isolates obtained from the animals included in this study, the AgNP concentration of 0.156 mg/mL inhibited 100% of the growth of 11 isolates. Two isolates showed a high susceptibility to AgNP, with 100% inhibition of growth up to the AgNP concentration of 0.02 mg/mL. The concentrations of 0.040 mg/mL and 0.080 mg/mL together demonstrated MIC_100_ for six isolates. Eight clinical isolates showed less susceptibility, with inhibition of 100% growth at a concentration of 0.312 mg/mL AgNP. Two isolates demonstrated MIC_100_ at a concentration of 0.625 mg/mL AgNP. The MBC_100_ ranged from 0.020 to 0.625 mg/mL, and only three isolates demonstrated the maximum MBC_100_ of 0.625 mg/mL (Supplementary Tables S1, S2).

### Evaluation of Surgical Wounds

**FIGURE 2:**
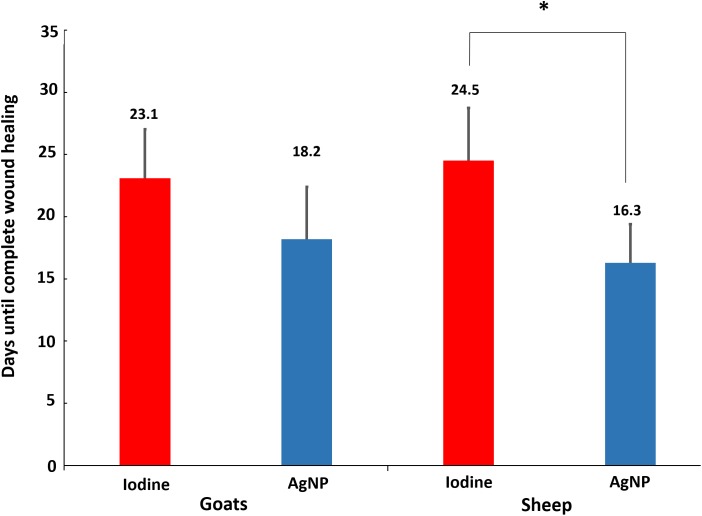
Average number of days needed for complete wound healing after the surgical procedure for the treatment of caseous lymphadenitis (CL). Twenty goats were used (10 treated with 10% iodine solution and 10 with AgNP ointment) and 9 sheep (four treated with 10% iodine solution and five with AgNP). The symbol “^∗^” indicates statistical difference with a *p* < 0.05 using the unpaired Student *t*-test.

As shown in Figure 2, surgical wounds of sheep treated with AgNP ointment took an average of 16.3 days to completely heal, while those treated with 10% iodine took an average of 24.5 days. *Post hoc* analysis revealed that this difference was significant (*p* < 0.05). The surgical wounds of nanoparticle-treated goats took approximately 18 days to completely heal, and those of 10% iodine-treated goats took an average of 23 days to completely heal. This result was not significantly different at *p* < 0.05. Associated with the faster wound healing, it could also be observed that the hair around the surgical wounds in the AgNP-treated animals grew faster (Supplementary Figure S2).

**FIGURE 3:**
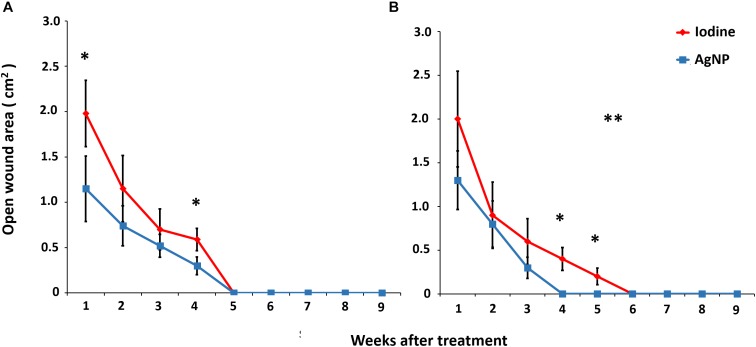
Areas of open surgical wounds in **(A)** 20 goats, 10 treated with 10% iodine solution and 10 with nanoparticle ointment, and **(B)** 9 sheep, 4 treated with 10% iodine solution, and five with AgNP ointment, after the CL surgical treatment. The means of each point were statistically compared using the unpaired *t* test and the “^∗^” symbol indicates statistical difference at *p* < 0. The healing curves are compared using the Wilcoxon signed rank and the symbol “^∗∗^” indicates statistical difference between the curves at *p* < 0.05.

Regarding the area of the surgical wounds (Figure 3), AgNP-treated goats presented a significant difference when compared to those treated with 10% iodine 1 and 4 weeks after the surgical procedure. The areas of the wounds were also smaller. The AgNP-treated sheep presented significant differences in surgical wound areas 4 and 5 weeks after removal of the caseous material when compared to those treated with 10% iodine (*p* < 0.05). The healing curve of AgNP-treated sheep was also significantly different from that of animals treated with 10% iodine at the Wilcoxon signed rank test (*p* < 0.05).

**FIGURE 4:**
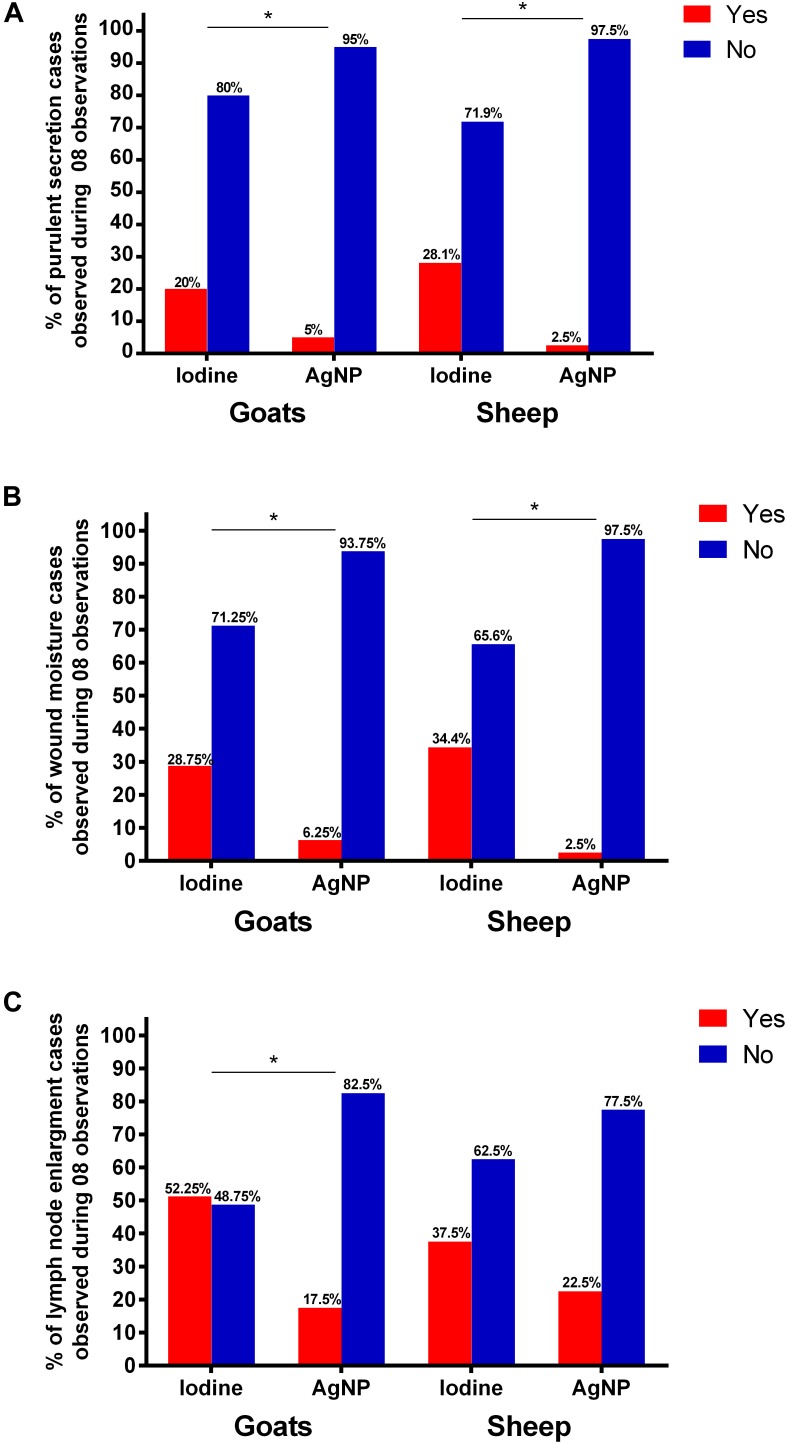
Presence of **(A)** purulent secretion and **(B)** moisture in surgical wounds an **(C)** detection of lymph node enlargement in animals treated with iodine or AgNP ointment after removal of CL lesions. The data express the number of events observed over the course of 8 weeks after the surgical procedure. The symbol “^∗^” stands for statistical difference between the iodine group and the group treated with nanoparticles at *p* < 0.05 using the chi square test.

Regarding the presence of purulent secretion in surgical wounds, Figure 4A expresses the number of events observed during 8 weeks after the surgical procedure. Goats treated with iodine demonstrated purulent secretion in their wounds in significantly more observations (20%) than those treated with the ointment (5%). In sheep, we observed pruritic discharges in the wounds of iodine-treated animals in 28% observations, whereas, in AgNP-treated sheep, this discharge was only observed once (2.5%). This difference was significantly different at the chi square test (*p* < 0.05).

Regarding the presence of moisture in the surgical wounds (Figure 4B), in both goats and sheep treated with AgNPs, humid wounds were observed in 6.25 and 2.5% of the observations, respectively. This was significantly different compared to animals treated with 10% iodine. Lymph node enlargement in goats treated with AgNPs was detected in 17.5% of the observations, while iodine-treated animals demonstrated lymph node enlargement in 52.25% of the observations, which was significantly different at the chi square test at *p* < 0.05 (Figure 4C). In sheep, there was no statistical difference in the occurrence of lymph node enlargement cases when the two treatments were compared.

### Clinical Post-surgical Evaluation of the Animals Treated With the AgNP Ointment and Iodine 10%

**FIGURE 5:**
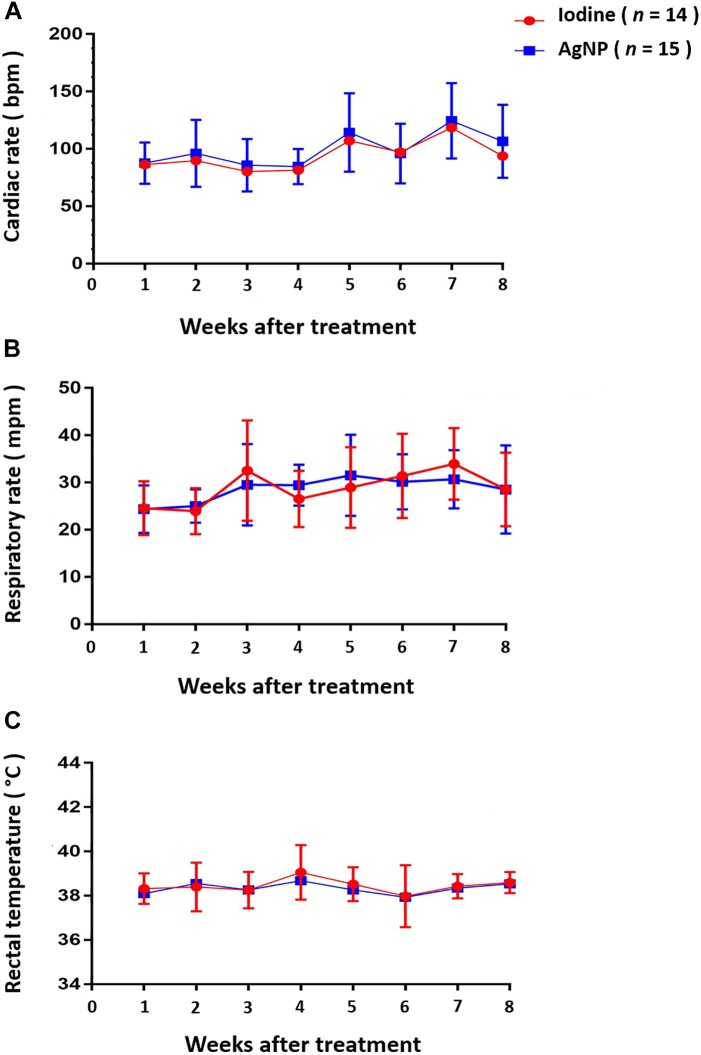
Clinical parameters of the animals that underwent the surgical procedure for CL lesions and treatment with iodine or AgNP. It were assessed the **(A)** cardiac rate (expressed in beats per minute), **(B)** respiratory rate (expressed in movements per minute), and **(C)** the rectal temperature (expressed in Celsius grades). The data are presented as the means of the groups at each moment of observation, and no statistical differences were seen between the groups at any evaluation point using the unpaired Student *t*-test (*p* < 0.05).

**FIGURE 6:**
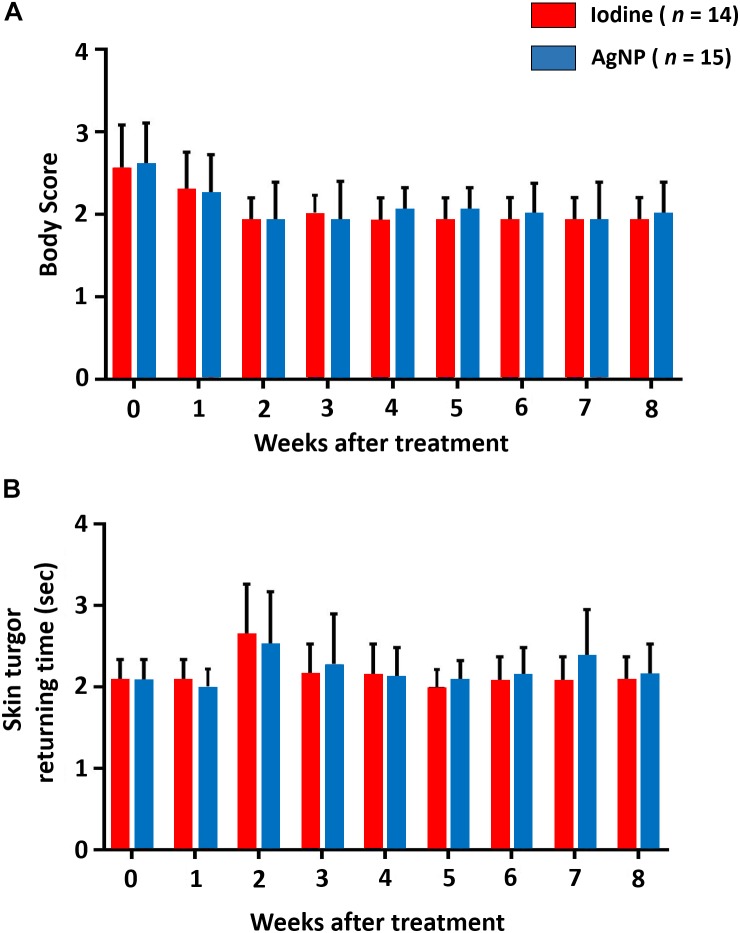
Determination of the **(A)** body score and the **(B)** skin turgor return time of animals treated with iodine or AgNP ointment. Results are presented as the means of each observation time for each group and the bars indicate their respective standard deviations. No statistical differences were seen between the groups at any evaluation point using the unpaired Student *t*-test.

The animals of this study were evaluated over a 2-month period, between May and July of 2017, in the municipality of Capim Grosso, Bahia State, Brazil. The average temperature in this location during this time of year is 28°C. The ambient temperature was constant during the course of this experiment. Figure 5 shows that, throughout the experiment, cardiac, and respiratory rate and rectal temperature presented little variation and there was no statistical significance between the two experimental groups at any time. Regarding the mean body scores, there was no statistical difference between the groups. Mean hydration levels, measured using the skin return time following the skin turgor test, were within normal limits, and no statistical difference was found between the two treatment groups (Figure 6). It is noteworthy to state that no clinical sign of dermatitis, photosensitivity or hypersensitivity was observed in animals treated with the AgNP ointment.

### Blood Cell Counts

**FIGURE 7:**
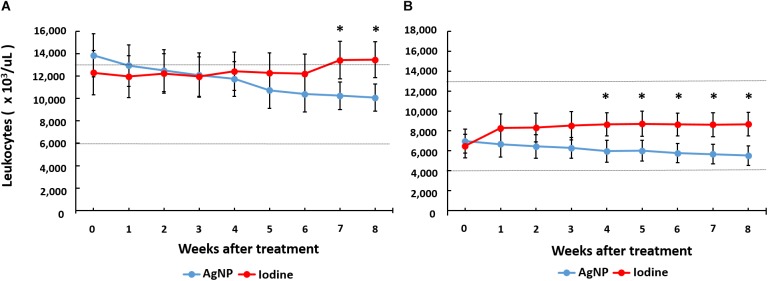
Total leukocyte counts of iodine or AgNP-treated goats **(A)** and sheep **(B)** after surgical removal of CL lesions. The means of each group at each point were compared using the unpaired Student *t*-test. The symbol “^∗^” represents a statistical difference at *p* < 0.05. The internal lines of each graph represent the lower and upper values of the reference value according to Weiss and Wardrop (2016).

In the evaluation of hemogram results (quantification of red blood cells, hemoglobin, globular volume, and mean globular volume), there was no statistically significant difference in either goats or sheep treated with AgNPs compared to those treated with iodine, and the values remained within the reference values described for small ruminants (Weiss and Wardrop, 2016). In the leukogram, the leukocyte count in iodine-treated goats was statistically higher than goats treated with AgNPs 5 to 8 weeks after the surgical treatment, with a statistical significance at the seventh and 8 week (*p* < 0.05). For sheep, the leukocyte count of iodine-treated animals was statistically higher from 4 weeks after the surgical procedure until the end of the observation period, as demonstrated in Figure 7.

### Immunological Assay

**FIGURE 8:**
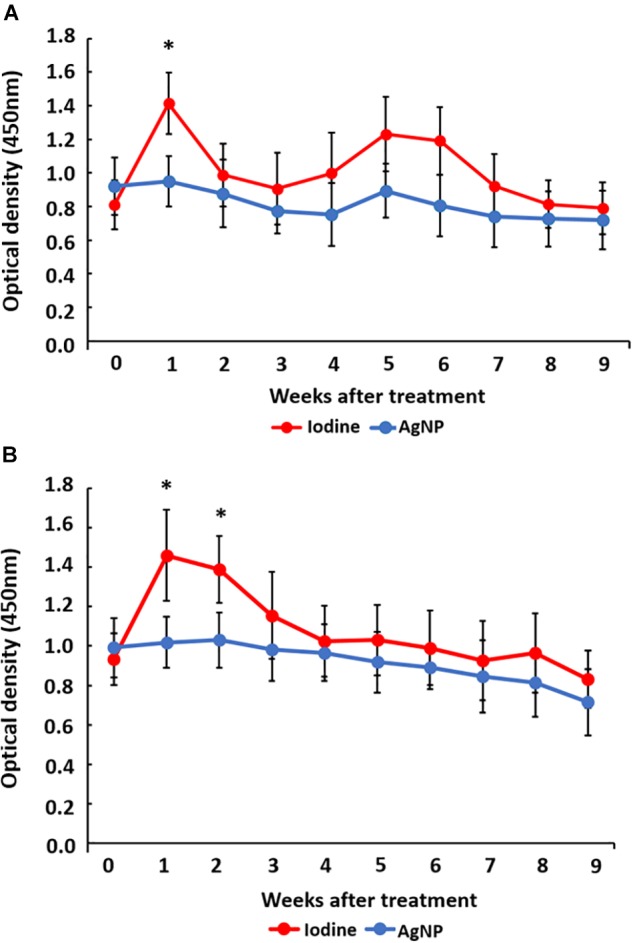
Detection of anti*-C. pseudotuberculosis*-specific antibodies in **(A)** goats and **(B)** sheep after surgical removal of CL lesions and treatment with iodine or AgNP ointment. The results are expressed as the means of optical densities per group by collection point. The bars represent the standard deviations. The means of each group at each point were compared using the unpaired Student *t*-test. The symbol “^∗^” represents a statistical difference at *p* < 0.05.

The detection of IgG immunoglobulins specific for *C. pseudotuberculosis* (Figure 8) demonstrated that, for both sheep and goats, production of specific antibodies peaked soon after the surgical intervention, but these levels normalized during the experiment. For goats, there was a higher production of *C. pseudotuberculosis* specific antibodies in the iodine-treated group in the first week, which was statistically different when compared to the AgNP-treated group. In sheep, differences between treatment groups occurred during the first 2 weeks after the surgical procedure, with specific antibody levels in the iodine-treated group higher than the AgNP-treated group, when values were compared by the unpaired Student *t*-test (*p* < 0.05).

## Discussion

The present study examines the effectiveness of AgNP-ointment as an alternative post-surgical treatment of CL in small ruminants. For a more complete verification of this application, verification of the clinical parameters of the treated animals, in addition to bacteriological tests, were conducted with standard strains, and clinical isolates of the bacterium.

The results of the broth microdilution susceptibility test confirmed that AgNPs demonstrated high efficacy in inhibiting the growth of three standard strains of *C. pseudotuberculosis* (T1, 1002, and FRC41) and clinical isolates collected from animals with caseous lesions, even at high dilutions. This finding agrees with studies showing the effectiveness of AgNPs against other bacteria, such as *Pseudomonas aeruginosa*, *Escherichia coli*, and *Salmonella* Typhi (Franci et al., 2015). Singh et al. (2013) tested bacterial strains of *Staphylococcus aureus* and *Streptococcus mutans* that exhibited resistance to β-lactam antibiotics and showed that the addition of AgNP reduced the minimal inhibitory concentration to the antibiotics and made the bacteria more susceptible to antibiotic treatment. In addition, it has been shown that AgNPs can be used as a topical drug owing to its high stability and low toxicity (Singh et al., 2013).

Regarding the lower susceptibility of the VD57 strain, it should be taken into account that VD57 is a highly virulent strain with a high replication rate and is well adapted to the culture conditions due to its long isolation time (Moura-Costa et al., 2008; Almeida et al., 2016). Although the relationship between virulence and susceptibility of *Corynebacteria* to antibiotics is not described, this relationship was observed in *Mycobacterium tuberculosis* (Rastogi et al., 1996), and in *Klebsiella pneumoniae* (Padilla et al., 2010).

It should be noted that, even though *in vitro* results demonstrated significant AgNP antimicrobial activity, *in vivo* CL treatment is difficult, because several characteristics of *C. pseudotuberculosis* infection and CL pathogenesis hamper the contact of drugs with the pathogen and provide some protection against certain antibiotics commonly used in the treatment of the disease. Among these factors, the presence of a thick fibrous capsule around the typical lesions, the caseous characteristic of the lesion, and even the intracellular nature of the organism during parts of the disease cycle can be seen (Williamson, 2001; Baird and Fontaine, 2007). Therefore, surgical excision of granulomatous lesions is still the most feasible option compared to systemic treatment with antimicrobial agents. Antibiotic drugs would be of greater importance in post-surgical treatment of the disease, since they inhibit environmental contamination (if the procedure is not done well and the lesion material continues to be released) or limit the spread of the pathogen in the animal after the lesion is broken. Thus, AgNP-containing ointment is possibly an effective treatment against CL, as it has good antimicrobial activity, a significant effect against bacteria resistant to antimicrobial agents, and its action is more concentrated on the physical aspects of the membrane, and therefore, is less likely to induce resistance (Apte et al., 2013; Rahisuddin et al., 2015; El-Batal et al., 2016; Halbus et al., 2017).

AgNP-treated animals demonstrated faster healing with a smaller area of the surgical lesion. It is worth noting that the wound healing process is complex and involves inflammation, granulation tissue formation, re-epithelization, and extracellular matrix remodeling (Chu et al., 2012; Heydarnejad et al., 2013; Chai et al., 2018). The clinical treatment of wounds remains a challenge in surgical procedures as the selected treatment should facilitate the healing process without producing harmful side-effects. Wound disinfection with 10% iodine solution is routinely used as the post-surgical treatment of LC but has several drawbacks; it is histotoxic and can disrupt the cicatrizing process (Burd et al., 2007; Santiago et al., 2013). This situation was observed in this study, as animals treated with iodine required a longer time for wound healing in both sheep and goats.

Recent studies have described the topical application of AgNPs for wound healing. Naraginti et al. (2016), who compared AgNPs with 1% soframycin, found that the healing time in rats, treated with gold nanoparticles, and AgNPs, was an average of 14 days after experimental surgery, whereas the group treated with 1% soframycin presented a delayed healing. Stojkovska et al. (2018) compared wound healing under AgNP treatment with commercial silver sulfadiazine cream and found that the AgNP-treated wounds took approximately 19 days to resolve, while the silver sulfadiazine-treated group took 21 days. Another recent study, described by Chai et al. (2018), observed, in humans, the resolution of AgNP-treated wounds in 17 days, 9 days sooner than the control treatment. The results obtained in the aforementioned studies align with the findings in the current study for both goats (18 days) and sheep (16 days).

Naraginti et al. (2016) also verified that the healing action of the nanoparticles in their study was most notable during the initial stages of wound healing, which resulted in a substantial reduction of open wound areas in the entire healing period. This effect was also observed in the current experiment; within the first week after the surgical procedure, the wound size in AgNP-treated animals was smaller than in the iodine-treated animals. This finding may be due to the fact that AgNPs present pronounced antimicrobial activity (Halbus et al., 2017). Their use in managing difficult-to-heal wounds can reduce the time required for tissue repair through anti-inflammatory effects and the prevention of wound colonization by opportunistic agents (Sandri et al., 2012; Stojkovska et al., 2018). Another study conducted by Silva et al. (2017) described that AgNPs induced a greater reduction in wound area by contraction, had low cytotoxicity, and promoted the growth of hair on the surface of the wound in a manner similar to that seen on the skin near the incision area. In our study, we were able to observe that wounds in animals treated with AgNPs not only healed faster, but also presented a faster hair growth.

Several studies report the healing potential of AgNPs, such as Galandáková et al. (2016), which demonstrated that AgNPs induce the release of a series of pro-inflammatory markers in several cell types, which accelerate healing. Stojkovska et al. (2018) stated that the decrease in wound area depends on contraction, as evidenced by Adibhesami et al. (2017) who found that AgNPs revert the inflammatory process in less time compared to antibiotics. In this study, not only did animals treated with iodine demonstrate more cases of pruritic exudate, but their wounds also maintained a moist environment ideal for bacterial growth. The increased purulent secretion observed in animals treated with iodine may be justified not only by the fact that iodine does not have an overall bactericidal action, but also because its use as an antiseptic has some disadvantages such as dermal irritation and a greater tendency to prolong inflammation (Iwasawa and Nakamura, 2003). It should be noted that animals in this study have not been treated for possible new CL lesions because it is believed that this would interfere with the immunological and hematological data of the animals. This non-isolation of the bacterium in new cases of lymphadenomegaly prevents the affirmation that further lymph node enlargement cases in animals treated with AgNPs may be due to *C. pseudotuberculosis* itself, however, it can be speculated that there was an inhibition of new infections by several bacterial agents that are sensitive to the AgNPs.

Several studies have shown that the temperature increase in different seasons may influence clinical parameters, such as cardiac and respiratory rates and rectal temperature (Marai et al., 2007). In our study, the temperature was constant for the duration of the experimental period, reducing environmental influences on these parameters. Similar values for the clinical parameters were observed across groups during the study period, and these parameters remained within the normal ranges (Riedi et al., 2018). This result reflects the fact that the treatment with AgNP-based ointment did not induce side-effects that would result in changes in these parameters. There were also no statistically significant differences in body and skin turgor return time scores. These findings indicate that the AgNP ointment did not induce physical side-effects, such as the development of hypersensitivity reactions, fever, or dermatitis. Pulit et al. (2011) cited that the contact of humans with AgNPs may cause adverse effects, and these different results may have been due to the different composition and thickness of the skin of small ruminants and humans. Another aspect to be considered is the size of the AgNPs, since Kang et al. (2017) described that 5 nm AgNPs amplify the clinical features of atopic dermatitis, and Park et al. (2011) found that 4 nm AgNPs presented a higher cytotoxic effect than AgNPs at high concentrations with 20 and 70 nm sizes. The AgNPs studied herein have an average size of 28 nm, and this characteristic can then be associated with a lower occurrence of adverse effects.

The results of the hemogram for both goats and sheep treated with AgNPs were within normal range, demonstrating that there were no toxic effects that could be expressed in hematological constituents. Similar results were observed in rabbits (Karavana et al., 2012), and humans (Chai et al., 2018), in which AgNPs showed low cytotoxicity. The higher leukocyte counts found in the animals treated with iodine, compared to those treated with AgNPs, are possibly due to iodine-related increases in levels of inflammation in ruminants due to tissue toxicity, and possibly due to fact that the topical application of nanoparticles are more effective in the suppression of inflammation (Naraginti et al., 2016). This has a consequent reduction of the presence of secondary bacterial infection in the small ruminants.

Immunity to *C. pseudotuberculosis* is attributed to humoral and cellular mechanisms (Lan et al., 1998). Humoral immunity is important in the fight against *C. pseudotuberculosis* since antibodies neutralize bacterial exotoxins, avoiding their dissemination in the host (Vale et al., 2016). The results obtained in this study regarding the detection of anti-*C pseudotuberculosis* specific antibodies, for both sheep and goats, demonstrated a higher production of these antibodies in the iodine-treated group of animals than in the AgNP-treated animals during the initial period of analysis. Goats and sheep that do not receive treatment after infection by *C. pseudotuberculosis* have a high humoral immune response for 70 days with peaks in the first weeks following removal of the lesions, and antibody titers beginning to decline after this period (Solanet et al., 2011). The lower titers of antibodies may be related to the absorption of AgNPs by the damaged skin, preventing the occurrence of antigenic stimuli by disseminated bacteria at the time of the surgical procedure. This absorption effect has already been described (Salomoni et al., 2017; Galbiati et al., 2018). Therefore, a lower infectious post-treatment load may warrant the lower titer of antibodies.

## Ethics Statement

This study was approved by the Committee on the Use of Animals in Scientific Experimentation of the School of Veterinary Medicine of the Federal University of Bahia (protocol number 35/2017).

## Author Contributions

DS, UM, LS, MM, TB, NS, and ND performed the experiments. UM, LS, and MM performed the data analysis. VA, LT, RM, and RP designed the experiments. RP, UM, and NS wrote the manuscript. VA, RM, and LT critically reviewed the manuscript. All authors contributed to manuscript revision, read and approved the submitted version.

## Conflict of Interest Statement

The authors declare that the research was conducted in the absence of any commercial or financial relationships that could be construed as a potential conflict of interest.
