# Supplementary material for: Biogenic Silver Nanoparticles as a Post-surgical Treatment for Corynebacterium pseudotuberculosis Infection in Small Ruminants
Source: Front Microbiol. 2019 Apr 24;10:824. doi: 10.3389/fmicb.2019.00824 (PMC6491793; doi:10.3389/fmicb.2019.00824)

Supplementary Figure S1. Surgical procedure used in this study for the excision of caseous lymphadenitis lesions in small ruminants. (A) Incision made with a #4 scalpel blade on the LC lesion; (B) drainage of the caseous material and collection using sterile containers; (C) cleaning of the wound using a sterile gaze; treatment of the surgical wound using (D) the AgNP-based ointment and (E) 10% iodine solution.

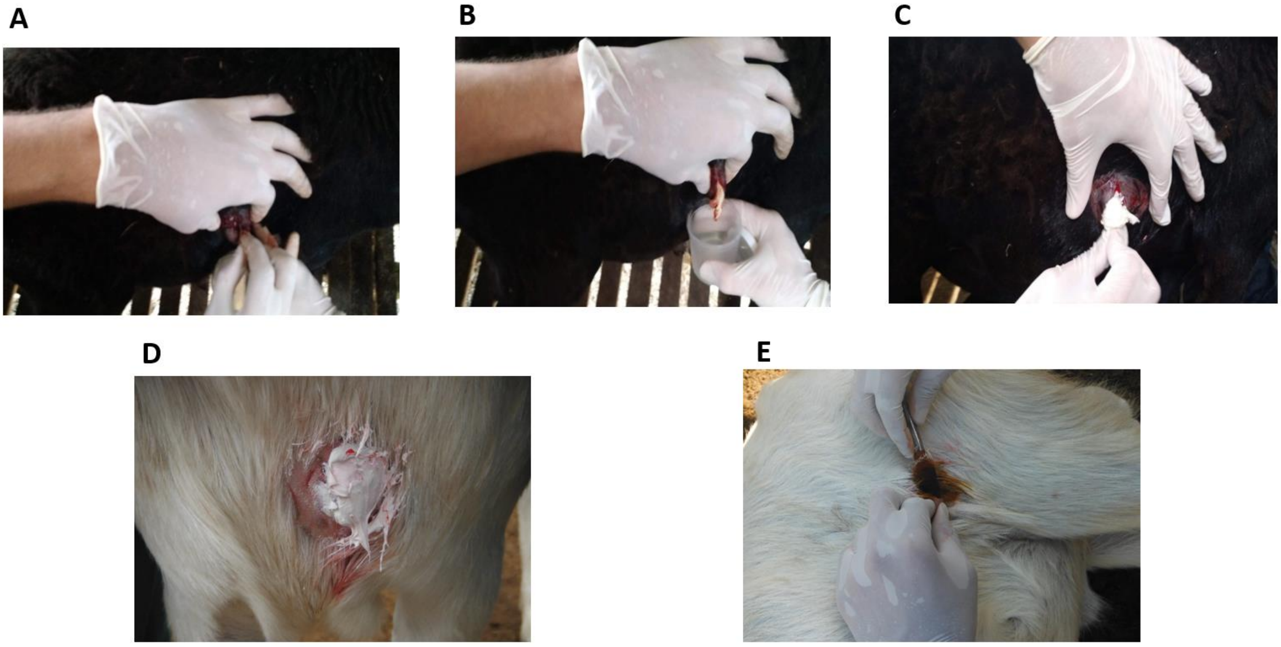

Supplement: Supplementary file 1 [file Image_1.pdf]
