# Supplementary material for: Biogenic Silver Nanoparticles as a Post-surgical Treatment for Corynebacterium pseudotuberculosis Infection in Small Ruminants
Source: Front Microbiol. 2019 Apr 24;10:824. doi: 10.3389/fmicb.2019.00824 (PMC6491793; doi:10.3389/fmicb.2019.00824)

Supplementary Figure S2. Aspects of the surgical wounds made in goats and sheep for the excision of caseous lymphadenitis lesions after the treatment with the AgNP-based ointment and with 10% iodine. (A) Surgical wound treated with 10% iodine, two weeks after the treatment, and (C) three weeks after the surgery. (B) Animal that had its surgical wound treated with the AgNP ointment, two weeks after the treatment, and (D) three weeks after the surgery.

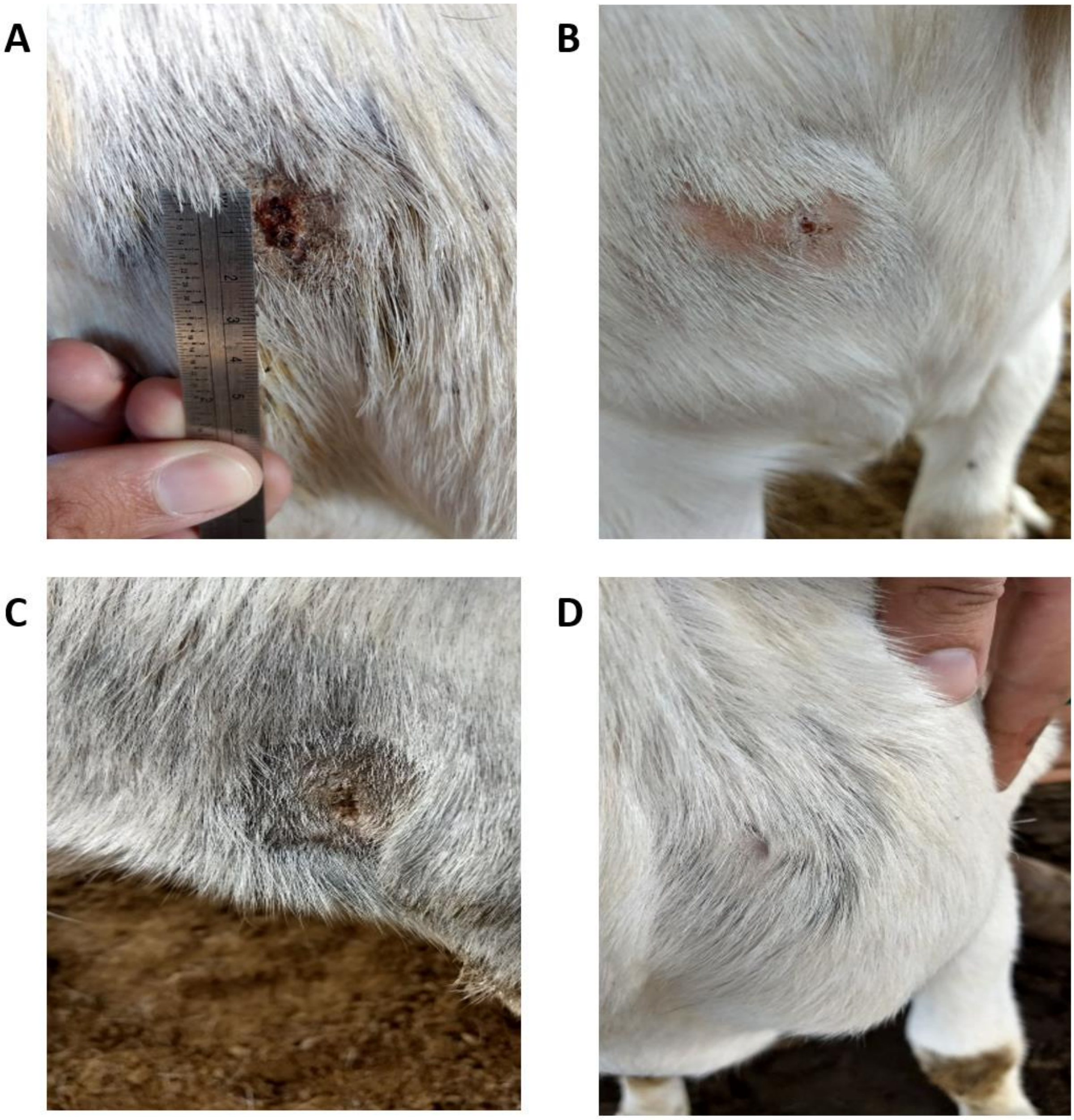

Supplement: Supplementary file 2 [file Image_2.pdf]
