# Supplementary material for: Biogenic Silver Nanoparticles as a Post-surgical Treatment for Corynebacterium pseudotuberculosis Infection in Small Ruminants
Source: Front Microbiol. 2019 Apr 24;10:824. doi: 10.3389/fmicb.2019.00824 (PMC6491793; doi:10.3389/fmicb.2019.00824)
Supplement: Supplementary file 3 [file Table_1.docx]

**Supplementary Table S1.** **Susceptibility to AgNP of *C. pseudotuberculosis* clinical isolates obtained from 20 goats that underwent excision of caseous lymphadenitis lesions.** A microdilution in broth assay was carried out with different AgNP dilutions (0.02 to 7.5 mg/mL). MIC_100_ is defined as the AgNP concentration in mg/mL where there was inhibition of 100% of bacterial growth, and MBC_100_ as the AgNP concentration where there was 100% of bactericidal action, in mg/mL.

| **Sample**  **ID** | | **MIC_100_**  **(mg/mL)** | | **MBC_100_**  **(mg/mL)** | | | | | |  |
| --- | --- | --- | --- | --- | --- | --- | --- | --- | --- | --- |
| 510 | | 0.312 | | 0.625 | | | | | |  |
| 512 | | 0.156 | | | | | 0.312 | | |  |
| 513 | | 0.156 | | 0.312 | | | | | |  |
| 514 | | 0.020 | | 0.040 | | | | | |  |
| 518 | | 0.156 | | 0.312 | | | | | |  |
| 522 | | 0.625 | | 0.625 | | | | | |  |
| 524 | | 0.156 | | 0.156 | | | | | |  |
| 527 | | 0.156 | | 0.156 | | | | | |  |
| 533 | | 0.020 | | 0.020 | | | | | |  |
| 541 | | 0.040 | | 0.080 | | | | | |  |
| 542 | | 0.312 | | 0.312 | | | | | |  |
| 543 | | 0.312 | | 0.312 | | | | | |  |
| 546 | | 0.312 | | 0.312 | | | | | |  |
| 563 | 0.040 | | | | 0.040 | | | |  |  |
| 568 | | | 0.156 | | | 0.156 | |  | | |
| 569 | | | 0.312 | | | 0.312 | |  | | |
| 570 | | | 0.156 | | | 0.156 | |  | | |
| 571 | | | 0.040 | | | 0.312 | |  | | |
| 583 | | | 0.312 | | | 0.625 | |  | | |
| 585 | | | 0.312 | | | 0.312 | |  | | |
