# Supplementary material for: Biogenic Silver Nanoparticles as a Post-surgical Treatment for Corynebacterium pseudotuberculosis Infection in Small Ruminants
Source: Front Microbiol. 2019 Apr 24;10:824. doi: 10.3389/fmicb.2019.00824 (PMC6491793; doi:10.3389/fmicb.2019.00824)
Supplement: Supplementary file 4 [file Table_2.docx]

**Supplementary Table S2.** **Susceptibility to AgNP of *C. pseudotuberculosis* clinical isolates obtained from nine sheep that underwent excision of caseous lymphadenitis lesions.** A microdilution in broth assay was carried out with different AgNP dilutions (0.02 to 7.5 mg/mL). MIC_100_ is defined as the AgNP concentration in mg/mL where there was inhibition of 100% of bacterial growth, and MBC_100_ as the AgNP concentration where there was 100% of bactericidal action, in mg/mL.

| **ID** | | **MIC_100_**  **(mg/mL)** | | **MBC_100_**  **(mg/mL)** | |  |  |
| --- | --- | --- | --- | --- | --- | --- | --- |
| 509 | | 0.156 | | **0.312** | |  |  |
| 517 | | 0.156 | | **0.312** | |  |  |
| 523 | | 0.156 | | **0.080** | |  |  |
| 526 | | 0.040 | | **0.312** | |  |  |
| 531 | | 0.040 | | **0.625** | |  |  |
| 532 | 0.625 | | **0.312** | |  | | |
| 562 | 0.312 | | **0.625** | |  | | |
| 564 | 0.156 | | **0.312** | |  | | |
| 567 | 0.080 | | **0.312** | |  | | |
